# RNAi targeting *Myzus persicae* (Hemiptera) stylet cuticle proteins reduces ZYMV transmission

**DOI:** 10.1016/j.virusres.2026.199784

**Published:** 2026-08-12

**Authors:** Jingfeng Liang, Amol Bharat Ghodke, Stephen J Fletcher, Narelle Manzie, Karl E Robinson

**Affiliations:** Queensland Alliance for Agriculture and Food Innovation, Centre for Horticultural Sciences, The University of Queensland, St Lucia, Australia

**Keywords:** RNA interference, Knockdown, Receptor, ZYMV, Stylet, *Myzus persicae*, Aphid

## Abstract

Aphids are one of the most important groups of agricultural pests due to their ability to transmit a wide range of plant viruses. The development of more sustainable and specific methods for plant virus control is an ongoing and active avenue of research. In this study, we demonstrate by feeding dsRNA to aphids via artificial diet assay, the knockdown of two *M. persicae* (green peach aphid) stylet cuticle proteins (*ST-1* and *ST-2*) implicated in the attachment and retention of the non-persistent, *Potyviridae* family member, zucchini yellow mosaic virus (ZYMV) (*Potyvirus cucurbitaflavitesselati*). We further enhanced *ST-1* and *ST-2* transcript knockdown by co-administering Nuclease dsRNA (NUC1/ -2_dsRNA) as an RNAi of RNAi approach, enhancing the survivability of orally co-delivered ST1 and ST2_dsRNA molecules against endonucleases in the aphid’s midgut. Functionally, we successfully show significant knockdown of the *ST1* and *ST2* gene upon co-administered with Nuc1_dsRNA translates to a significant reduction of virus particle retention of up to 84% and 90%, respectively in aphids. Particle reduction translated to a 60 – 73% reduction in the infection rate when zucchini plants were challenged with treated aphids. This study demonstrates the successful application of an RNAi of RNAi approach to significantly disrupt the virus-vector interaction effectively reducing virus transmission to plants and laying a solid foundation towards effective multi-stage life cycle targeting with RNAi-based viricidal applications.

## Highlights

• The green peach aphid is one of the most destructive insect pests.
• The green peach aphid is a primary vector for plant viruses.
• dsRNA against stylet cuticle and gut nuclease proteins reduced virus retention.
• Particle reduction translated to significant reduction in plant infection rate.
• Application of RNAi significantly disrupted the virus-vector interaction.

## Graphical abstract

**Figure:**
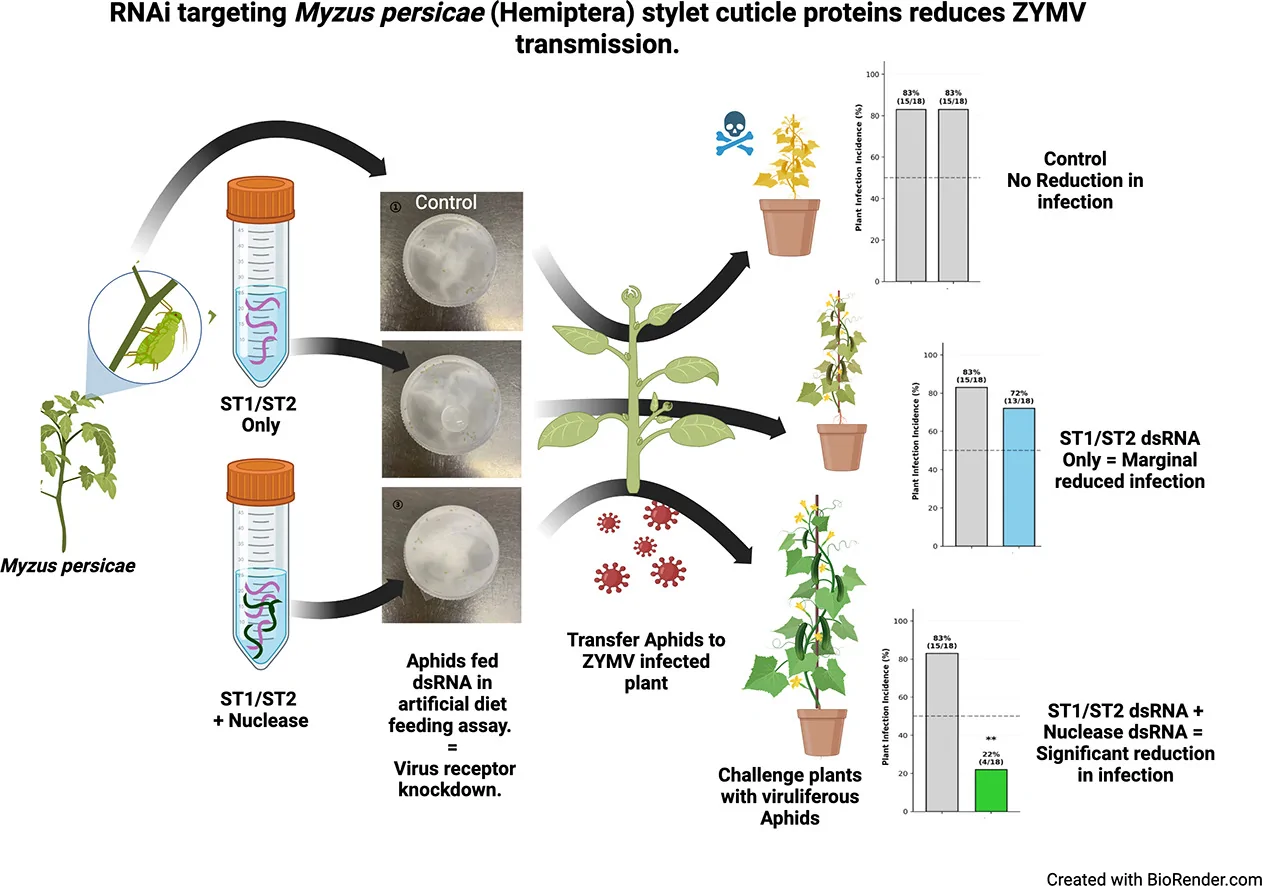

## 1 Introduction

Aphids are a large group of small piercing-sucking insects of the order Hemiptera encompassing approximately 4400 species that are distributed worldwide (Hulle et al., 2010). Over 200 aphid species are recognised as vectors for plant viruses (Blackman & Eastop, 2000) with the model aphid species, *Myzus persicae*, (Ali et al., 2006) a generalist feeder able to predate numerous plant species and vector over 200 known plant viruses (Carr et al., 2020).

The interaction between virus and vector is a complex and dynamic relationship with plant viruses being broadly classified into two major groups termed ‘non-persistent’ or ‘persistent’. The latter group also encompassing semi-persistent viruses representing an intermediate group of plant viruses. These designations are dependent on the retention of the respective virus at specific anatomical sites within the vector and the capacity of the virus to persist, replicate, and be transmitted by their vector (Gray & Banerjee, 1999; Ng & Zhou, 2015; Hogenhout et al., 2008).

The dynamics of non-persistent and persistent virus particle retention is exemplified by the amount of time required for virus uptake, translocation to the site of retention, and inoculation / transmission of particles to the plant. The acquisition access period (AAP), for successful virus uptake and retention of non-persistent viruses is in the order of seconds, contrasting hours required for persistent viruses. Likewise, the ‘inoculation access period’ (IAP) dictates the time required for inoculation of the virus from the vector to the plant, with only minutes required for non-persistent viruses or the several days required for persistent viruses (Hogenhout et al., 2008).

Approximately, 75% of known plant viruses, including one of the largest groups of plant viruses, *Potyviridae*, are transmitted in a non-persistent manner (Carr et al., 2020; Deshoux et al., 2018). Potyviral AAP and IAP is in the order of seconds and minutes respectively with viral particles retained via association with stylet cuticle proteins within the maxillary stylet tip of the aphid acrostyle (Gray & Banerjee, 1999; Martín et al., 1997; Powell, 2005). Nonpersistent viruses from different genera have various virion-stylet attachment strategies and although the mechanism of non-persistent virion attachment is not fully resolved, two attachment strategies have been defined.

**Fig. 1:**
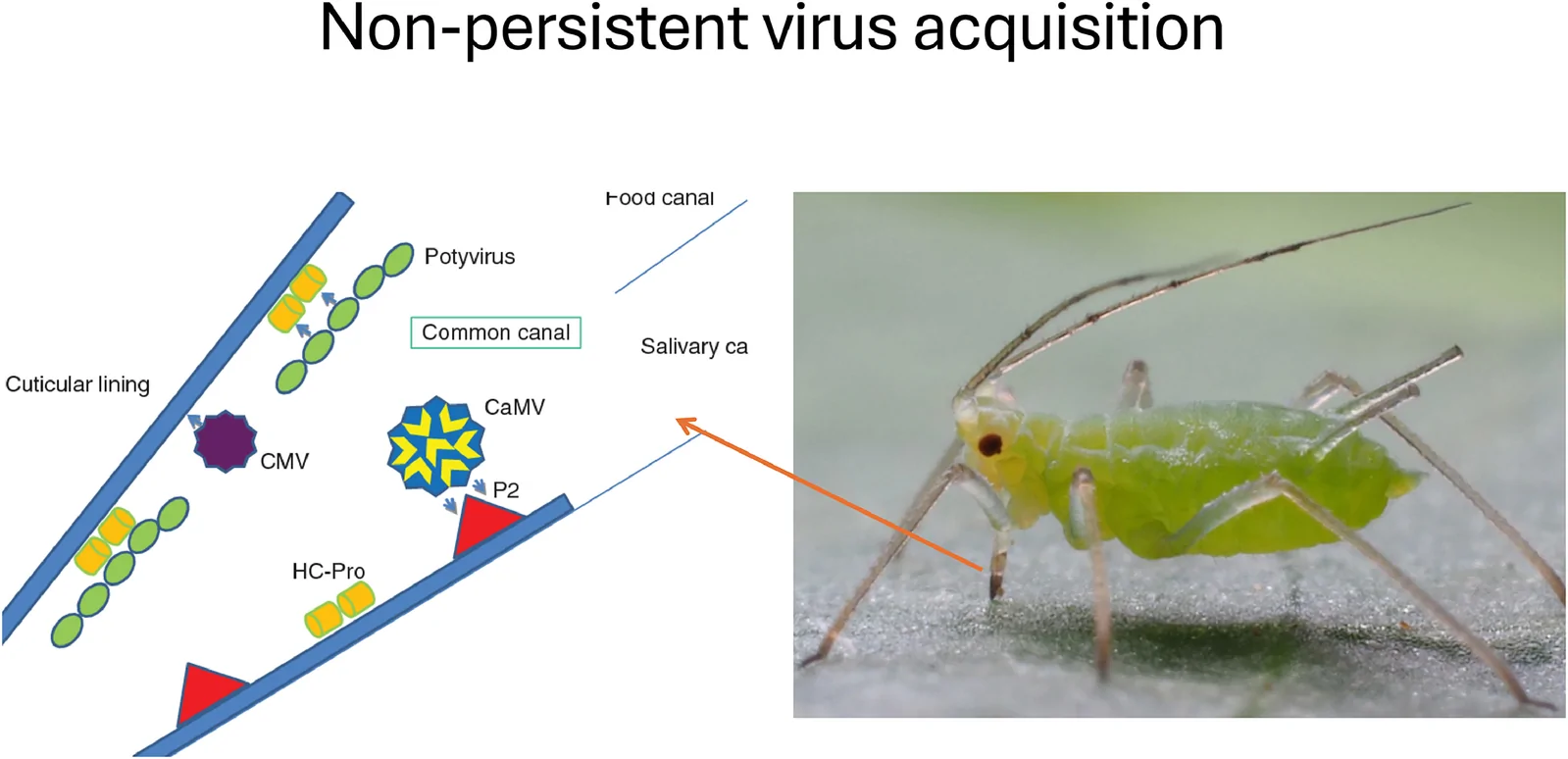
**Non-persistent plant virus acquisition by aphids**. Aphids retain non-persistent virus particles via specific interactions between stylet cuticle proteins and virus coat proteins. Two types of attachment strategy are recognised, direct attachment as exploited by cucumber mosaic cucomovirus (CMV) or indirect attachment via the use of a bridging ‘helper component’ protein (HC-Pro). *Potyviridae* family members encode the multifunction HC-Pro whereas cauliflower mosaic virus (CaMV) encodes the HC-pro designated P2. Schematic of virus attachment modified from (Fereres & Raccah, 2015). Aphid image courtesy of Scott Bauer, Wikimedia Commons (https://commons.wikimedia.org/wiki/File:Myzus_persicae.jpg)[Accessed 10^th^ Nov 2025].

For *Potyvirus* genus members, a ‘helper strategy’ is employed where critical, reversable interactions between the aphid stylet cuticle protein(s) and a virus encoded multifunction ‘bridging’ protein, designated ‘helper component protein’ (HC-Pro), and the potyviral nucleocapsid occur (Fig. 1) (Maia et al., 1996; Pirone & Blanc, 1996). Contrasting a ‘helper’ attachment strategy, several virus genera use a ‘direct attachment’ strategy. In this instance, the nucleocapsid interacts with the respective cuticle protein directly. For example, this attachment strategy is utilised by the *Bromoviridae* family members and exemplified by the type species cucumber mosaic virus (CMV) of the *Cucumovirus* genus (Ng & Falk, 2006) (Fig. 1).

The critical interactions between virus particle and the stylet cuticle protein, irrespective of the attachment strategy, presents an opportunity to develop alternative control strategies against non-persistent viruses by challenging the ability of virus particles to be retained, and transmitted by vectors. Prior research efforts have identified several proteins located within the proximal end of the stylet, that interact with viral nucleocapsid and bridging proteins of *Potyvirus cucurbitaflavitesselati*, zucchini yellow mosaic virus (ZYMV) (Dombrovsky et al., 2007). Furthermore; Liang et al. (2017) has also defined several candidates with the interaction of four cuticle proteins in the retention of CMV (Liang & Gao, 2017).

Seminal research by Webster et al. (2018) identified two cuticular proteins within the stylet of *Acyrthosiphon pisum* and *M. persicae*, designated stylin-01 (ST1) and stylin-02 (ST2) (Webster et al., 2018). Studying cauliflower mosaic virus (CaMV) (*Caulimovirus tessellobrassicae*) attachment interaction, where CaMV utilises the bridging strategy via the virus encoded Protein 2 (P2), it was shown that RNA interference (RNAi) mediated knockdown of ST1, but not ST2, by orally delivered small interfering RNA (siRNA) resulted in a significant titre reduction in CaMV virus particles retained within the stylet of *M. persicae*. This reduction in particle retention translated to a decrease in transmission efficiency and plant infection incidence and suggested ST1 is critical for CaMV virion attachment, retention, and transmission and is the primary protein mediating attachment of CaMV via P2 to the stylet. It follows that if the interaction between the viral nucleocapsid and the protein mediating particle attachment can be disrupted or negated, via RNAi applications, a potential method to mitigate plant virus transmission within a crop can be achieved.

RNAi is a sequence-specific nucleic acid-based mechanism that is highly conserved in eukaryotes that functions in post transcriptional regulation (Elbashir et al., 2001) and antiviral defence (Rosa et al., 2018). RNAi has been exploited as a functional genomics tool in numerous pest insects via the introduction of long double-stranded RNA molecules (dsRNAs) or siRNAs either through ingestion, injection, or topical application (Jain et al., 2020). As a crop protection tool, RNAi has been utilized as a genetic modification (GM) / transgenic technology termed Host Induced gene silencing (HIGS) against infection or insect predation for over thirty years (Fitchen & Beachy, 1993; Stark & Beachy, 1989; Wilson, 1993).

Antiviral strategies for plant viruses have dominantly targeted their vectors using chemical insecticides or HIGS via GM of the respective crop. However, the long-term application of insecticides induces ever-increasing rates of insecticide resistance in vectors and potential adverse effects on the environment. Whereas HIGS via GM of fruit and fibre crops faces regulatory hurdles, long lead in times, and community aversion limiting wide-spread use of transgenic technologies. These hurdles have driven the search for alternative control strategies. Most recently, the concept of RNAi by topical application termed, spray induce gene silencing (SIGS) to induce specific knockdown of virus (Kaldis et al., 2018; Konakalla et al., 2016; Konakalla et al., 2019; Tenllado & Diaz-Ruiz, 2001; Tenllado et al., 2004) or insect genes disrupting significant physiological and homeostatic functions, such as metabolism, growth, osmoregulation and immunity to cause mortality has been gaining momentum (Bilgi et al., 2017; Yan et al., 2020; Xie et al., 2022; Ghodke et al., 2025).

In either aphids or plants, exposure of dsRNA activates the endonuclease enzyme DICER to digest the dsRNAs into 21-24bp siRNAs. These siRNAs are then assembled into the RNA Inducing Silencing Complex (RISC) to direct RISC to the target homolog. Here, the enzymatic component of RISC, Argonaute (AGO), digests the target transcript to ultimately, inhibit the formation of target proteins (Elbashir et al., 2001). Application of RNAi via topical application requires a non-replicative, dsRNA molecule homologous to critical genes of the pest or pathogen to be applied to plants to induce a transgenic-like albeit transient response (Tenllado & Diaz-Ruiz, 2001; Tenllado et al., 2004). Extending the concept of SIGS to disrupt the virus-vector interaction, to reduce the ability of the aphid vector to retain non-persistent viral particles, to reduce vector transmission efficiency and therefore reduce infection incidence in plants is plausible.

However, one major limitation of dsRNA feeding is the survivability of ingested dsRNA in the presence of gut nucleases (Ghodke et al., 2019; Tayler et al., 2019). Rapid degradation of dsRNA by gut nucleases, either extra-orally or upon ingestion directly influences the efficiency of RNAi in insects and has been a confounding factor limiting the magnitude and consistency of RNAi gene knockdown applications (Ghodke et al., 2019; Christiaens et al., 2014). RNAse activity has been reported in several families including hemipterans *Myzus persicae* (Ghodke et al., 2019), *Acyrthosiphon pisum* (Cao et al., 2018; Christiaens et al., 2014; Jaubert-Possamai et al., 2007), and others (Jain et al., 2021; Tayler et al., 2019), including members of the Tephritidae family of fruit flies (*Bactrocera* tryoni) (Tayler et al., 2019), beetles within the Order Coleptera (Colorado potato beetle) (Spit et al., 2017), grasshoppers within the family Acrididae (Song et al., 2019) and the most recalcitrant to RNAi application, members of the Order Lepidoptera (Guan et al., 2018; Jain et al., 2021; Tayler et al., 2019). To overcome this limitation, strategies such as increasing dsRNA concentration, structurally modifying dsRNA/siRNA molecules (Abe et al., 2012), or most promising, co-targeting nuclease genes (Ghodke et al., 2019; Tayler et al., 2019; Webster et al., 2018) as an RNAi of RNAi approach have been shown to improve RNAi efficacy.

In this study, we first evaluated the efficacy of orally delivered dsRNA via artificial diet assay in reducing the expression of *ST1* and *ST2* genes in the aphid *M. persicae*. Functionally, we investigate whether RNAi induced knockdown of *ST1* and/or *ST2* genes can limit the retention of ZYMV (*P. cucurbitaflavitesselati)* particles in aphids. Here we found that reduction was achieved and comparable to prior studies in a research context but in general, was suboptimal for potential deployment as a crop protection strategy. To further bolster the effectiveness of ingested dsRNA targeting *ST1* and *ST2* we co-administered dsRNA molecules targeting the gut nucleases of *M. persicae* defining a significant knockdown of ST1 and ST2, a reduction in virus particle retention and infection rate in plants. In addition, we provide tentative evidence for the ST2 cuticle protein to be the primary attachment site for ZYMV. Ultimately, we show *in planta* the knockdown of *ST1* or *ST2* gene expression can significantly reduce the transmission efficiency of ZYMV and the infection rate in *Cucurbita pepo* (zucchini) plants.

## 2 Material and methods

### 2.1 Plant, virus, and insect maintenance

*Cucurbita pepo* var. Regal Black (*C. pepo* (RB)) was used for all virus propagation and aphid (*Myzus persicae*, green peach aphid) colony maintenance. Plants were grown from seed in 100 mm pots and maintained under glasshouse conditions ∼ 25/18°C day/night temperature, and ∼60% relative humidity until required.

*Potyvirus cucurbitaflavitesselati* isolate WA C19-001 (ZYMV-C19), kindly provided by Dr. Craig Webster (DPIRD, Western Australia), was originally isolated from infected melon crops in Carnarvon, Western Australia in 2019 (Clarke et al., 2020). ZYMV-C19 was propagated in *C. pepo* (RB) via mechanical inoculation using infected *Cucurbita moschata* var. Kent leaf tissue (∼1 g) homogenized in 5 mL of cold 10 mM sodium phosphate buffer (pH 7.4) supplemented with 1 g L⁻¹ sodium sulphite (Sulistyowati et al., 2004). Leaves of *C. pepo* (RB) were dusted with carborundum abrasive, gently rubbed with the viral lysate, and rinsed with water. Virus propagation was repeated every 30 days under glasshouse conditions. ZYMV-C19 infection was confirmed using DAS-ELISA (Agdia, USA; #SRA 27200) (Poty-ELISA) for all propagation and experimental samples following manufacturers protocol with OD values used for scoring a positive sample threshold set at three times OD of the negative plant control reading.

The aphid colony was established in 2019 from ∼20 wild *M. persicae* individuals collected from *Taraxacum officinale* (common dandelion) in Ipswich, Queensland Australia (27° 36′ 56" S, 152° 45′ 40" E). Aphids were starved for ∼16 hours at 4°C before being transferred to *C. pepo* (RB) seedlings. Plants were observed for any overt viral infection symptoms with both plants and aphids screened at 30 days post-placement using poty-ELISA and DAS-ELISA for cucumber mosaic cucomovirus (CMV) (*Cucumovirus CMV*) (Agdia, USA; #SRA 44501), tomato spotted wilt virus (TSWV) **(***Orthotospovirus tomatomaculae*) (Agdia, USA; SRA 39300), and bean common mosaic virus (BCMV) (*Potyvirus phaseovulgaris*) (Agdia, USA; SRA 46000) as per manufactures protocol. The aphid colony was maintained in vented BugDorm-4 cages (47.5 cm³) within climate-controlled growth cabinets set to a 16:8-hour light/dark photoperiod, 26/18°C day/night temperature, and 70% relative humidity.

### 2.2 dsRNA synthesis

Linear DNA templates for *in vitro* dsRNA synthesis of ST1-dsRNA, ST2-dsRNA, NUC1_dsRNA and NUC2_dsRNA were amplified from aphid genomic material by two successive rounds of PCR using the respective chimeric primer pairs (Table 1) generating respective dsRNA effectors (**Supplementary data 1**). Primers incorporated a 5’ T7 polymerase promoter sequence followed by ∼20 bp of homologous sequence of the respective aphid gene. All molecular biology reagents were sourced from Meridian Science, Australia unless stated otherwise. Reactions (50 µL) contained 10x reaction buffer, 5U MyTaq polymerase, 0.4 μM of each respective reverse/forward chimeric primer and 0.2 ng µL^-1^ cDNA generated from total RNA extracted from apterous *M. persicae* aphids at instar stage 3 to 5. PCR cycling conditions were: initial denaturation at 95°C for 1 min, followed by 30 cycles of 95°C for 15 s, 54°C for 15 s and 72°C for 10 s, final extension of 72°C for 5 min and a completion hold at 12°C. Primary amplicons were resolved on 0.8% TAE gel for 1 hr at 90V and purified using the Wizard SV gel purification kit (Promega, USA). Purified amplicons were used as template for second round PCR reactions using the same components and conditions as above. Purified template DNA (∼1.5 µg) was used for *in vitro* synthesis of dsRNA using a T7 Hi Scribe kit (New England Biosciences, Australia) as per manufacturers protocol.

**Table 1:** *In vitro* transcription template and real time PCR primer pairs.

Primer Name	Sequence 5’ - 3’^a.^	Efficiency^.b^
Mp_ST1_T7_F	TAATACGACTCACTATAGGGagaagaagtcaggtacttgaagg	
Mp_ST1_T7_R	TAATACGACTCACTATAGGGgtgctcaccgtcttactggt	
Mp_ST2_T7_F	TAATACGACTCACTATAGGGgcccaaggtcccgaatctaag	
Mp_ST2_T7_R	TAATACGACTCACTATAGGGtcagctgggattggtggtga	
Mp_NUC1_T7_F	TAATACGACTCACTATAGGGtcctccgacgcgttggtcac	
Mp_NUC1_T7_R	TAATACGACTCACTATAGGGtgttgccgtacagctctttg	
Mp_NUC2_T7_F	TAATACGACTCACTATAGGGgatggccttgtggccaataat	
Mp_NUC2_T7_R	TAATACGACTCACTATAGGGgctaaatgcccacggtcaaa	
ZYMV(WA)_HC-Pro_F	TAATACGACTCACTATAGGGtcacatcagcgtgtgtctccca	
ZYMV(WA)_HC-Pro_R	TAATACGACTCACTATAGGGcagtttgatgcgctgtgttggc	
Mp_ST1_qPCR_F	gtccaagattcagcacccag	93.5%
Mp_ST1_qPCR_R	ctcgaatgggattgctggtg	
Mp_ST2_qPCR_F	cacaaaccaccaaaatgcag	100.9%
Mp_ST2_qPCR_R	gcttttgcgtaaccgacttc	
Mp_RPL7_qPCR_F	tgccggagtctgtactcaaa	109.9%
Mp_RPL7_qPCR_R	cacgcgttctttacgttcct	
ZYMV(WA)_HC-Pro_qPCR_F	gcaacacaggcaactcagaa	R^2^ = 0.9991
ZYMV(WA)_HC-Pro_qPCR_R	gctgtttcagagccaagtcc	

^a. Upper case lettering denotes T7 polymerase recognition sequences.^

^b. Efficiency determination refers to^**^Supplementary Data 2^**

### 2.3 Reverse transcription real time quantitative PCR

Assessment of relative ST1, ST2, and Ribosomal protein L7 (RpL7) gene expression in aphids and virus titre was by RT-qPCR as per previous (Ghodke et al., 2025). Briefly, target-specific gene primers (Table 1) for use in one-step RT-qPCR were designed in Primer 3 (V0.4.0) (http://bioinfo.ut.ee/primer3-0.4.0/). Primers for assessing aphid stylet gene knockdown were designed to amplify a region 5’ adjacent to sequence targeted by the respective dsRNA effectors. Primers for assessing virus titre were designed to amplify a 160bp region (1378 – 1537bp) of the HC-pro gene of ZYMV-C19. qRT-PCR reactions were performed using CFX 96 Realtime thermocycler (Bio-Rad) and Bio-Rad CFX Maestro 1.1 (version 4.1.2433.1219) software. Relative target gene expression was quantitated from TriSure (Meridian Biosciences, AU) extracted and TURBO DNase I (Invitrogen) treated total aphid RNA with the following components and conditions. Each 20 µL reaction contained; 2 x Sensifast No ROX One-Step Mix, 0.4µM of respective forward and reverse qPCR primer (Table 1), 0.2µL reverse transcriptase, 0.4µL RNase Inhibitor and 10 ng of DNase I treated template RNA. Cycling conditions included 10 min at 45°C and 2 min at 95°C followed by 35 cycles of 95°C for 5 seconds, 60°C for 10 sec and 72°C for 5 sec with melt curve analysis conducted post-amplification. Relative gene expression quantitation between control and treatment groups utilised the 2^-ΔΔCt^ (Livak & Schmittgen, 2001) method by normalising the expression of *M. persicae* target genes against the reference gene RpL7 (Kang et al., 2017; Lü et al., 2018).

Absolute quantification of virus titre in ZYMV-C19 challenged zucchini plants from total RNA extracted from respective treatment groups was via comparison to a standard curve dilution series (10^-1^ - 10^5^) (**Supplementary Data 3**). Briefly, the standard curve was established using a 10-fold serial dilution (10 ng μL^-1^ to 10⁻⁴ ng μL^-1^) of *in vitro* transcribed HC-Pro_dsRNA as per method for dsRNA synthesis above using primers ZYMV(WA)_HC-Pro_F / ZYMV(WA)_HC-Pro_R (Table 1). For each dilution, 1 μL was added to a 10 μL qPCR reaction with primers HC-Pro_qPCR_F/ HC-Pro_qPCR_R (Table 1) with the same components and conditions as above, and four technical replicates per dilution. The mean Ct value was plotted against the log₁₀-transformed copy number of HC-Pro dsRNA per reaction, which was determined using the following formula (**EQ1**).

$$\mathrm{EQ}1:\;\text{Copy}\;\text{number}=\;\frac{\text{Quantity}\;\text{of}\;\text{RNA}\;\left(\text{ng}\right)\;x\;\text{Avogadro}\;\text{constant}:6.022\;x\;10^{23}}{\text{RNA}\;\text{length}\;\left(552\;\text{bp}\right)\;x\;1\;x\;10^{9}\;x\;\text{average}\;\text{weight}\;\text{of}\;\text{dsRNA}\;\text{per}\;\text{bp}\;\left(660\;g/\text{mol}\right)}$$

To quantify ZYMV particle retention in aphids, 10 ng of aphid total RNA was added to a 10 μL qPCR reaction with the same components and conditions as above, and four technical replicates per sample. The mean Ct values for each sample was calculated by substitution into the equation of the standard curve (y = -3.2959 + 50.003) (EQ1), to generate the log10-transformed copy number of HC-Pro gene of each sample, which was used to calculate the copy number for each sample.

### 2.4 Orally delivered dsRNA for aphid stylin gene knockdown

*Stylin-dsRNAs to reduce stylin gene expression:* Artificial diet (AD) feeding assays were performed to assess the efficacy of orally delivered ST1_ or ST2_dsRNA to knockdown aphid Stylin gene expression. Cohorts of ∼25 aphids were collected in 25mm petri dishes (Corning, USA) and fed with respective treatments; Negative controls (filter sterilised 30% sucrose-only and nonspecific non-target (NS)_dsRNA (**Supplementary Data 1**)), and respective ST1_dsRNA and ST2*_*dsRNA targeting the Stylin genes 1 and 2, respectively. The dsRNA treatments were orally delivered through a Parafilm membrane sachet at a final concentration of 300 ng μL^-1^ in 200 μL of 30% sucrose. AD assays were repeated three times, and each treatment group had four biological replicates. Aphids were maintained in a growth cabinet with the same conditions as those for rearing colonies. After 72 hours of feeding, 10 aphids were collected from each biological replicate, total RNA extracted and the relative expression of *ST1* and *ST2* was assessed by RT-qPCR.

### 2.4.1 Enhancement of stylin gene knockdown with gut nuclease-targeting dsRNA

To further optimise Stylin gene knockdown, the co-administration of Nuclease_dsRNA with Stylin_dsRNA, as an RNAi of RNAi approach was used to reduce nuclease transcript abundance and increase the protection of the ST1 and ST2_dsRNA from premature nuclease degradation when ingested. This assay was set up and repeated as previous, with controls (30% sucrose and NS_dsRNA) and treatments ST1 and ST2_dsRNA at 300 ng μL^-1^ supplemented with nuclease-dsRNAs (Nuc1_dsRNA and/or Nuc2_dsRNA) at a final concentration of 150 ng μL^-1^ each (total dsRNA concentration 450 ng μL^-1^). The treatment groups were ST1 or ST2_dsRNA + Nuc1_dsRNA, ST1 or ST2_dsRNA + Nuc2_dsRNA (total dsRNA concentration 450 ng μL^-1^), and ST1 or ST2_dsRNA + Nuc1 + Nuc2_dsRNA (total dsRNA concentration 600 ng μL^-1^). Each assay was repeated three times, with each treatment having four biological replicates. After 72 hours of feeding, ∼10 aphids were harvested, total RNA extracted and the relative expression of respective gene transcripts assessed by RT-qPCR. (NS_dsRNA control was kept at 300 ng μL^-1^ irrespective of the total concentration of dsRNA in treatment groups since validation of NS_dsRNA as a robust off-target control across all three concentrations was independently tested - refer **Supplementary Data 4**)

#### 2.4.2 ZYMV particle retention in aphid stylet

To evaluate whether the knockdown of either Stylin genes can lead to reduction in the ZYMV viral particle retention, AD feeding of respective dsRNAs was performed as per the previous assays including controls (30% sucrose and NS_dsRNA) and the treatment groups; ST1_dsRNA [300 ng μL^-1^], ST1_dsRNA + Nuc1_dsRNA [total 450 ng μL^-1^], ST2_dsRNA [300 ng μL^-1^] and ST2_dsRNA + Nuc1_dsRNA [total 450 ng μL^-1^]. Each assay was repeated three times, and each treatment had four biological replicates. After 72 hours of feeding, ∼10 aphids were transferred to detached ZYMV-C19 infected *C. pepo* (RB) leaves for up to 30 minutes AAP to acquire virus. The titre of ZYMV-C19 particles was assessed by RT-qPCR.

#### 2.4.3 Mitigation of aphid transmitted ZYMV infection

Here we aimed to define if a reduction in ZYMV virion retention via the knockdown of aphid Stylin genes can reduce ZYMV transmission efficiency and infection incidence in plants. As above, aphids were fed via AD controls (30% sucrose and NS_dsRNA) and treatments ST1_dsRNA [at 300 ng μL^-1^], ST1_dsRNA + Nuc1_dsRNA [total 450 ng μL^-1^], ST2_dsRNA [at 300 ng μL^-1^] and ST2_dsRNA + Nuc1_dsRNA [total 450 ng μL^-1^], and subsequently transferred to ZYMV-infected leaves, for 30 min AAP to acquire virus. Five viruliferous aphids were then transferred to clip cages on healthy zucchini seedlings and maintained overnight (18 hours) under growth cabinet conditions. Aphids were dispatched by treatment of plants with Imidacloprid at 0.05 g L^-1^ and plants subsequently maintained under glasshouse conditions. The infection status of challenged plants was assessed by Poty-ELISA at 10 days post-challenge. This assay was repeated three times, and each treatment had six biological zucchini seedling replicates.

### 2.5 Graphs and statistical analysis

All graphs and statistical analyses were performed using GraphPad Prism 10 for Windows (GraphPad Software, Boston, MA, USA; www.graphpad.com). Prior to analysis, normality was assessed using the Shapiro–Wilk test and homogeneity of variance using Levene’s test. As these assumptions were not met for several datasets, heteroscedasticity-robust methods were applied throughout. Welch’s ANOVA followed by Dunnett’s T3 post-hoc test was used for the continuous gene-expression and viral-retention data; Dunnett’s T3 does not assume equal variances and controls the family-wise error rate across all pairwise comparisons.

#### 2.5.1 Stylin-dsRNAs to reduce stylin gene expression

**Fig. 2:**
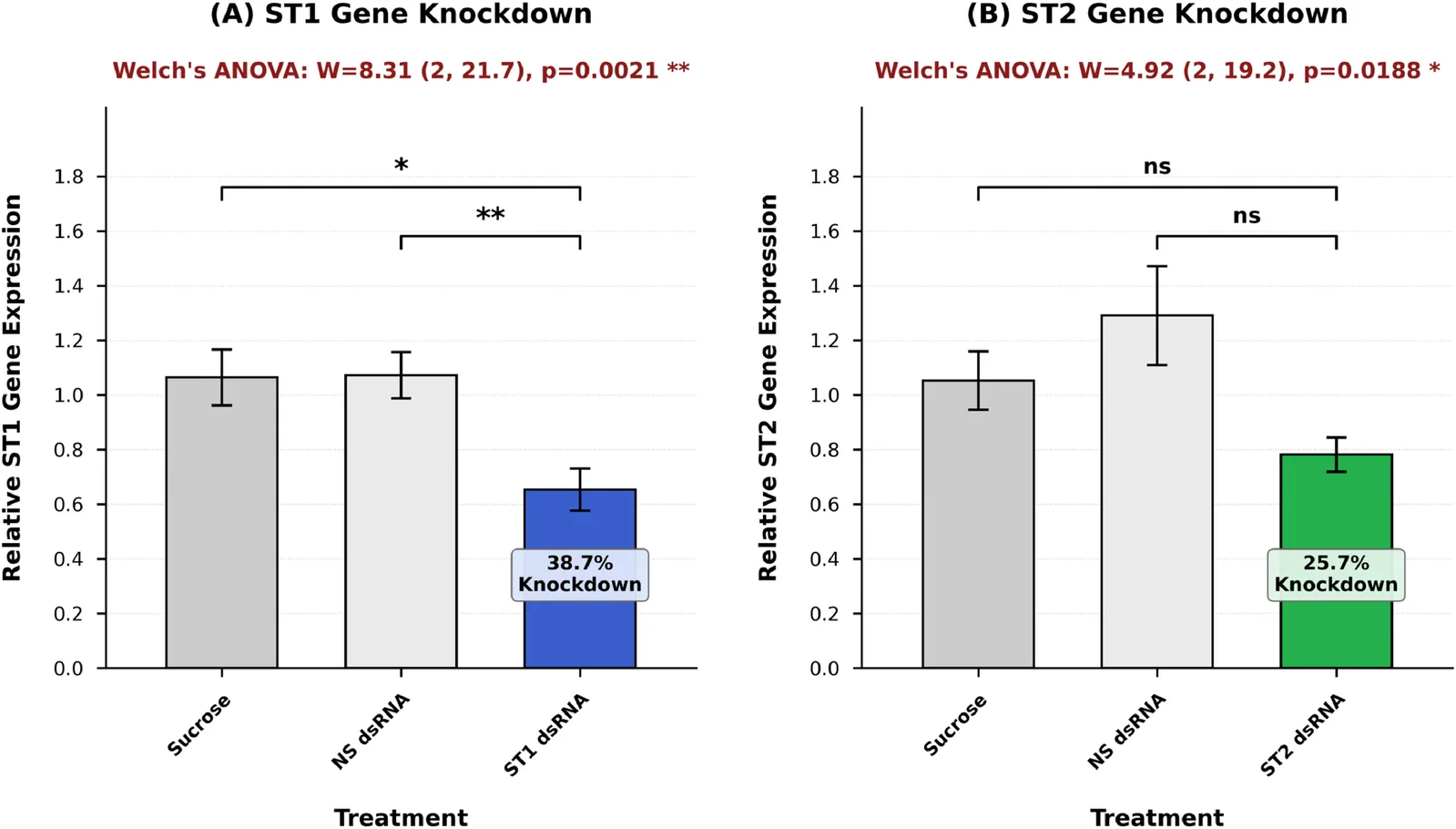
**Relative expression of *M. persicae* Stylin gene (*ST1* and *ST2*).** Panels (A) and (B) display *ST1* and *ST2* relative gene expression following artificial diet feeding for 72 hours with ST1 or ST2 dsRNA (300 ng/µL). Controls include sucrose (baseline) and non-specific (NS) dsRNA. Expression was quantified by RT-qPCR and normalised to the housekeeping gene RpL7; values are normalised expression ratios rather than fold-change relative to sucrose, and the sucrose means therefore differ slightly from 1.0. Data are presented as mean ± SEM (n=12) with knockdown percentages, calculated relative to the sucrose control, labelled within the treatment bar. Statistical comparisons were performed using Welch's ANOVA followed by Dunnett's T3 post-hoc multiple comparison tests; brackets denote the groups compared. ST1 dsRNA reduced *ST1* expression by 38.7 % (Welch's ANOVA W=8.31, p=0.0021; Dunnett's T3 versus sucrose p=0.0124, versus NS dsRNA p=0.0040). For *ST2*, Welch's ANOVA indicated a treatment effect (W=4.92, p=0.0188) but no pairwise comparison reached significance after correction (25.7 % reduction; versus sucrose p=0.1204, versus NS dsRNA p=0.0535). ns, not significant; *p<0.05; **p<0.01.

The effectiveness of orally delivered ST1_dsRNA and ST2_dsRNA in reducing target gene expression was evaluated by comparing relative gene expression among the sucrose control, non-specific dsRNA (NS_dsRNA) control and gene-specific dsRNA treatments (n = 12 per treatment group; Fig. 2). Welch’s ANOVA was used to test for an overall treatment effect within each gene, followed by Dunnett’s T3 post-hoc test for pairwise comparisons between treatment groups.

#### 2.5.2 Enhancement of stylin gene knockdown with gut nuclease-targeting dsRNA

**Fig. 3:**
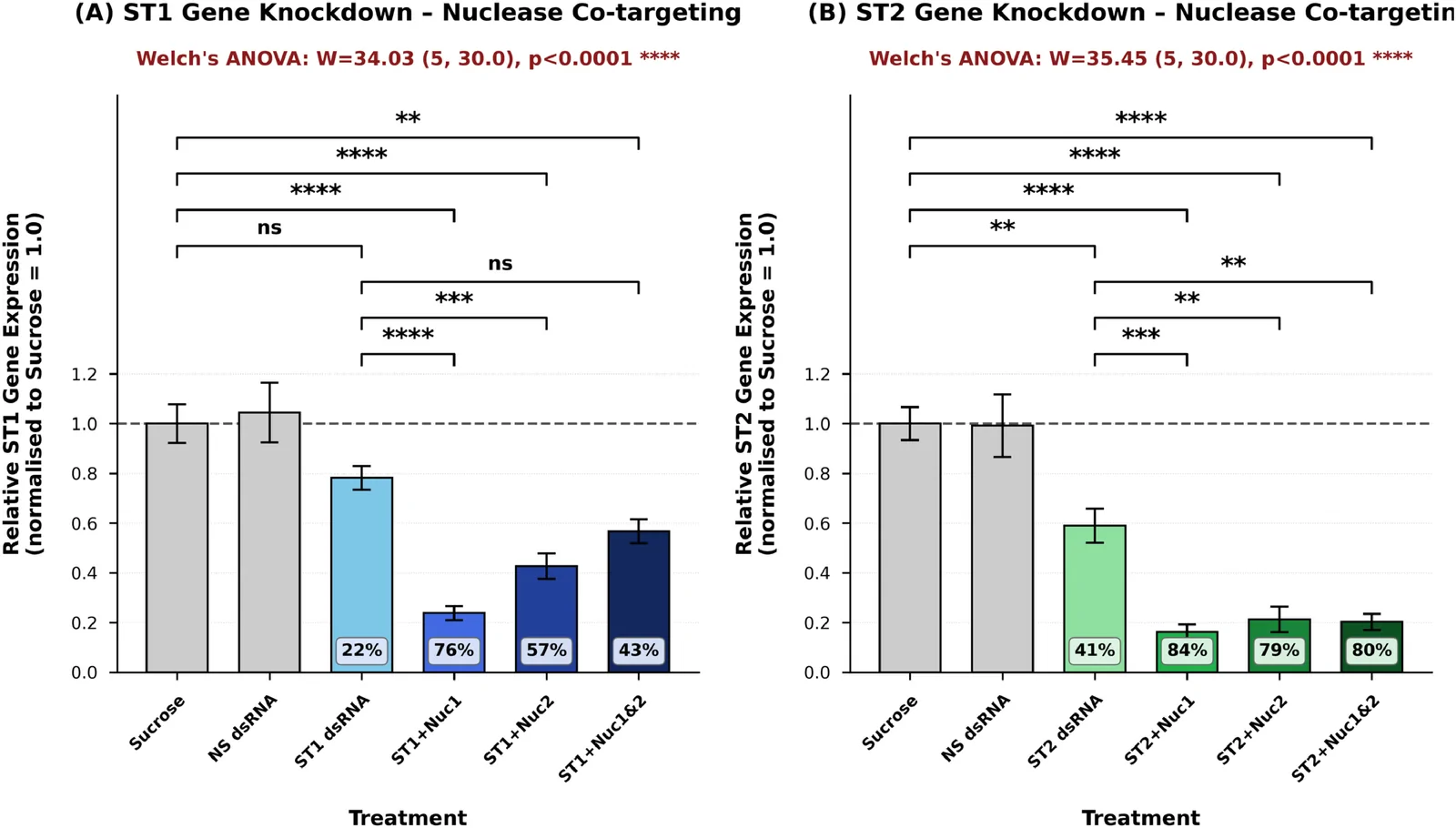
**Relative expression of aphid (*M. persicae*) stylin gene (*ST1* and *ST2*) with Nuclease supplementation.** Panels (A) and (B) display *ST1* and *ST2* relative gene expression following artificial diet feeding for 72 hours with ST1 or ST2 dsRNA (300 ng/µL) alone or co-administered with Nuc1 dsRNA (150 ng/µL), Nuc2 dsRNA (150 ng/µL), or both Nuc1+Nuc2 dsRNA (150 ng/µL each). Controls include sucrose (baseline) and non-specific (NS) dsRNA. Expression was quantified by RT-qPCR, normalised to the RpL7 housekeeping gene, and expressed relative to the sucrose control mean (dashed line = 1.0). Data are presented as mean ± SEM (n=12) with knockdown percentages, calculated relative to the sucrose control, labelled within each gene-specific dsRNA bar. Statistical comparisons were performed using Welch's ANOVA followed by Dunnett's T3 post-hoc multiple comparison tests; brackets denote the groups compared, with the lower tier comparing each nuclease co-treatment against the gene-specific dsRNA alone and the upper tier comparing each treatment against the sucrose control. Nuclease co-administration increased *ST1* knockdown from 22 % with dsRNA alone to 76 % (Nuc1), 57 % (Nuc2) and 43 % (Nuc1+Nuc2), and *ST2* knockdown from 41 % to 84 %, 79 % and 80 %, respectively (Welch's ANOVA: *ST1* W=34.03, p<0.0001; *ST2* W=35.45, p<0.0001). ns, not significant; **p<0.01; ***p<0.001; ****p<0.0001.

The effects of ST1_dsRNA or ST2_dsRNA supplemented with nuclease-targeting dsRNA (Nuc1_dsRNA and/or Nuc2_dsRNA) on gene knockdown were evaluated across six treatment groups per gene (sucrose control, NS_dsRNA, gene-specific dsRNA alone, and gene-specific dsRNA co-administered with Nuc1_dsRNA, Nuc2_dsRNA, or Nuc1+Nuc2_dsRNA; n = 12 per treatment group; Fig. 3). Welch’s ANOVA was used to test for an overall treatment effect, and following a significant omnibus result Dunnett’s T3 post-hoc test was applied for all pairwise comparisons.

#### 2.5.3 ZYMV particle retention in the aphid stylet

**Fig. 4:**
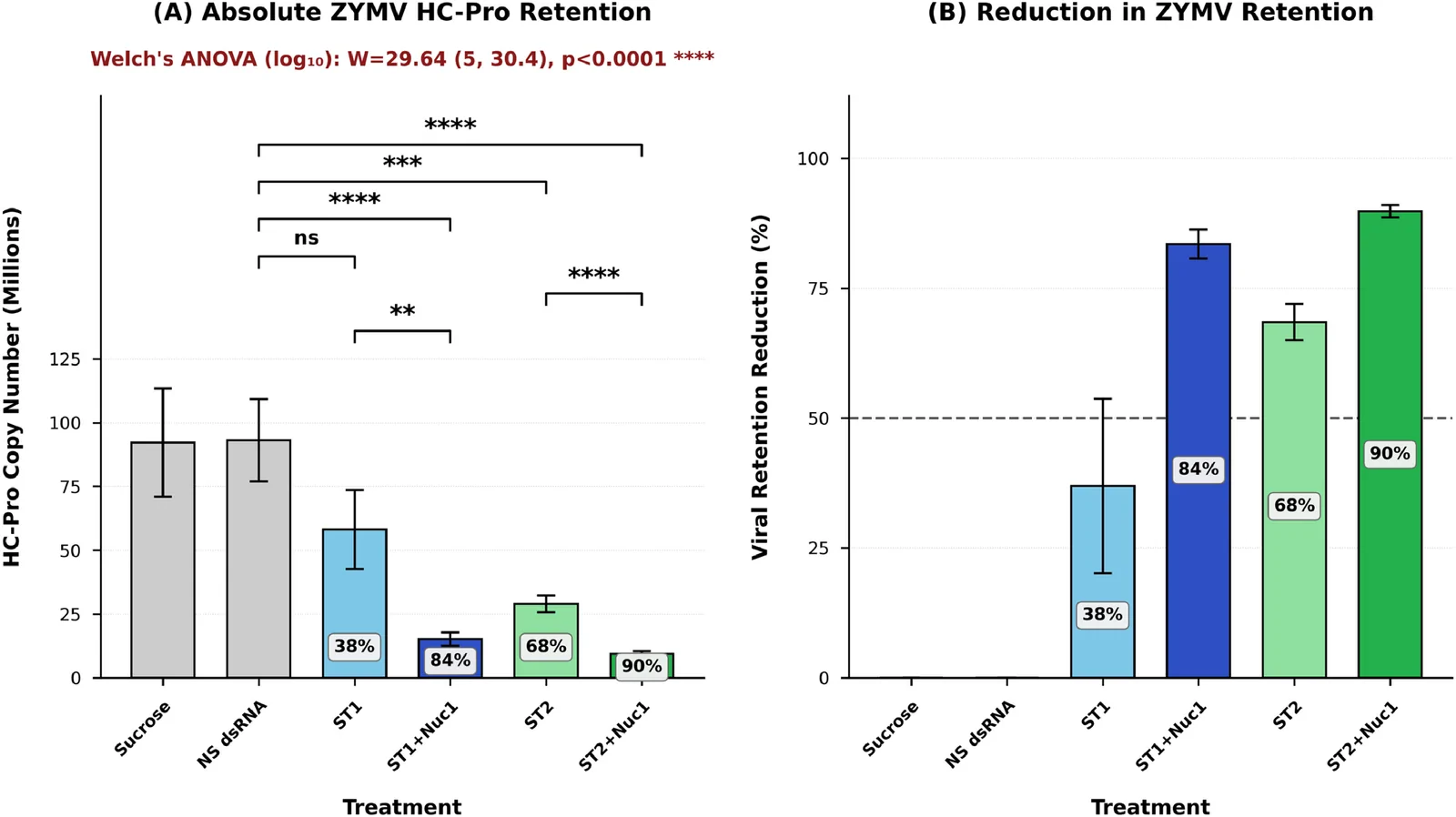
**Impact of ST1 and ST2 Gene Knockdown on ZYMV Particle Retention in *Myzus persicae.*** Panels (A) and (B) display ZYMV HC-Pro copy number (A, absolute values in millions) and the corresponding reduction in viral retention relative to the sucrose control (B), in aphids treated with ST1 or ST2 dsRNA ± Nuc1 co-administration. Aphids were fed artificial diet containing the treatments for 72 hours, then transferred to ZYMV-infected plants for 30 minutes to acquire virus. HC-Pro was quantified by RT-qPCR. Controls include sucrose (baseline) and non-specific (NS) dsRNA. Data are presented as mean ± SEM (n=12 per group), with reduction percentages labelled within the treatment bars of both panels; in panel (B) the two control groups are plotted at the zero baseline for reference. Because HC-Pro copy number spanned two orders of magnitude and showed strongly unequal variances between groups (Bartlett's test p<0.0001), all statistical analysis was performed on log₁₀-transformed copy numbers using Welch's ANOVA followed by Dunnett's T3 post-hoc multiple comparison tests, consistent with the approach used in Fig. 2, Fig. 3. Brackets in panel (A) denote the groups compared: the lower tier compares each nuclease co-treatment against the corresponding dsRNA-alone treatment, and the upper tier compares each treatment against the NS dsRNA control, which controls for the delivery of dsRNA itself. Panel (B) presents the same data expressed as percentage reduction; statistical comparisons are shown in panel (A) only, to avoid duplicate annotation of identical tests. Welch's ANOVA indicated a highly significant treatment effect (W=29.64; df=5, 30.4; p<0.0001). Relative to the NS dsRNA control, ST1+Nuc1, ST2 and ST2+Nuc1 significantly reduced HC-Pro retention by 84 % (p<0.0001), 68 % (p=0.0005) and 90 % (p<0.0001) respectively, whereas ST1 alone (38 %) did not (p=0.3717). Nuclease co-administration significantly enhanced the effect of both dsRNAs (ST1 versus ST1+Nuc1, p=0.0013; ST2 versus ST2+Nuc1, p<0.0001). The two control groups did not differ from one another (sucrose versus NS dsRNA, p=0.9991). ns, not significant; **p<0.01; ***p<0.001; ****p<0.0001.

ZYMV particle retention was quantified by measuring HC-Pro copy number in aphids using absolute-quantification RT-qPCR across six treatment groups (n = 12 aphids per treatment group; Fig. 4). HC-Pro copy number spanned approximately two orders of magnitude with strongly unequal variances between groups (Levene’s and Bartlett’s tests, *P* < 0.01), consistent with an approximately log-normal distribution, and copy-number values were therefore log_10_-transformed prior to analysis. Welch’s ANOVA was applied to the log_10_-transformed data, followed by Dunnett’s T3 post-hoc test for pairwise comparisons against the NS_dsRNA control and between each gene-specific dsRNA treatment and its nuclease co-administration.

#### 2.5.4 Mitigation of aphid-transmitted ZYMV infection

**Fig. 5:**
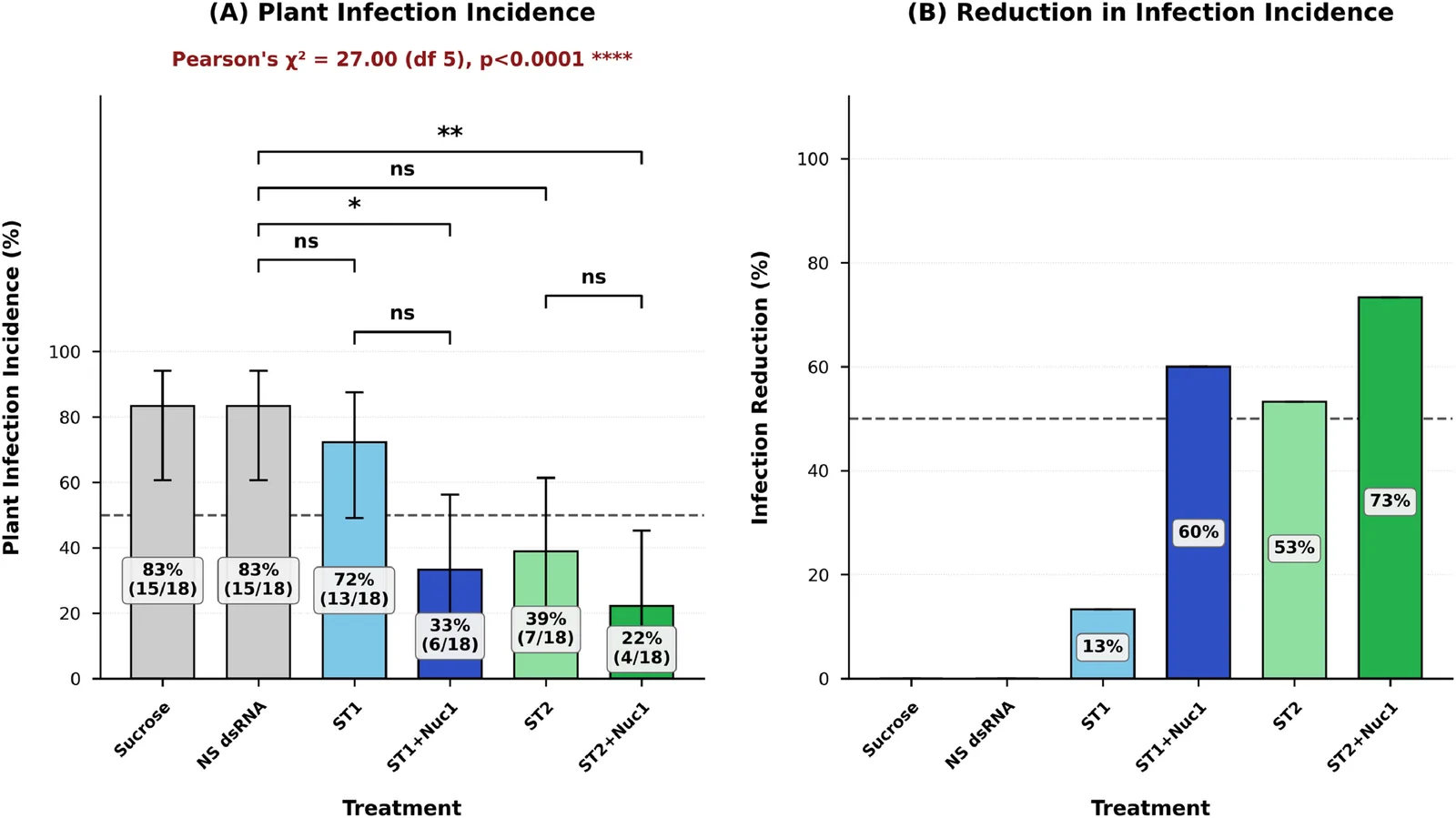
**Impact of *ST1* and *ST2* gene knockdown in aphids on ZYMV transmission to zucchini.** Aphids were fed artificial diet containing the treatments for 72 hours, transferred to ZYMV-infected leaves for a <30 minute acquisition access period, and five viruliferous aphids were then placed on each healthy zucchini seedling for an 18 hour inoculation access period. Aphids were removed by imidacloprid treatment and infection status was determined by DAS-ELISA at 10 days post-challenge (n=18 plants per treatment), scoring plants as infected above an OD₄₀₅ threshold of 3 times the **(XXXX**). (A) Plant infection incidence, shown as the percentage of plants infected with the number infected per group given within each bar; error bars are Wilson 95 % confidence intervals. Treatment effects were assessed by Pearson's χ² test on the 2 × 6 contingency table (χ²=27.00, df=5, p<0.0001), followed by pairwise Fisher's exact tests with Holm correction across the six pre-specified comparisons shown. Brackets denote the groups compared: the upper tier compares each treatment against the NS dsRNA control, and the lower tier compares each nuclease co-treatment against the corresponding dsRNA-alone treatment. ST1+Nuc1 and ST2+Nuc1 significantly reduced infection incidence relative to the NS dsRNA control (33 % and 22 % versus 83 %; p=0.0297 and p=0.0037), whereas ST1 alone (72 %, p=0.941) and ST2 alone (39 %, p=0.061) did not reach significance after correction. (B) The same data expressed as the reduction in infection incidence relative to the 83 % control baseline; the dashed line marks the 50 % threshold adopted as a practical disease-management benchmark. Panel (B) is a descriptive rescaling of panel (A) and is plotted without error bars, since the reduction is a deterministic function of the incidence proportions whose uncertainty is shown in panel (A); control groups are at the zero baseline by definition. Statistical comparisons are likewise shown in panel (A) only, to avoid duplicate annotation of identical tests. ns, not significant; *p<0.05; **p<0.01.

Plant infection incidence (number of infected plants out of 18 per treatment group) was analysed as a 2 × 6 contingency table using Pearson’s χ² test to determine whether infection status was associated with treatment (Fig. 5A). Following a significant omnibus result, pairwise comparisons were made using Fisher’s exact test, with the Holm–Bonferroni sequential-rejection procedure applied across the pre-specified comparisons to control the family-wise Type I error rate (α = 0.05). Infection status was assigned from DAS-ELISA positivity threshold OD >0.205. Panel B (Fig. 5B) presents the same data as the percentage reduction in infection incidence relative to the control and is a descriptive rescaling of Panel A; no separate statistical test was applied to it.

#### 2.5.5 Significance notation

Throughout, *P* < 0.05 was considered statistically significant, and significance is denoted as * *P* < 0.05, ** *P* < 0.01, *** *P* < 0.001, **** *P* < 0.0001; ns, not significant. Knockdown and retention-reduction percentages reported in Fig. 2, Fig. 3, Fig. 4 express the treatment group mean relative to the sucrose control mean.

#### 2.5.6 Sample size and replication

Gene-knockdown assays (Fig. 2, Fig. 3) and the ZYMV particle-retention assay (Fig. 4) each comprised n = 12 biological replicates per treatment group. The transmission and infection-incidence assay (Fig. 5) was performed as three independent experiments, each with six biological zucchini seedling replicates per treatment group, yielding 18 plants per treatment in total. Technical replication comprised 3–4 RT-qPCR reactions for gene-expression assays and 4 reactions for viral quantification.

## 3 Results

### 3.1 Stylin-dsRNAs to reduce stylin gene expression

To evaluate the effectiveness of orally delivered dsRNA in reducing Stylin gene (*ST1* and *ST2*) expression, aphids fed on artificial diets (30% sucrose solution) supplemented with respective NS, ST1 or ST2_dsRNA (300 ng μL^-1^) showed the relative expression levels of both *ST1* and *ST2* genes was modestly reduced by ∼38.7% (*P* < 0.05) and 25.7% (ns), respectively when compared to sucrose controls (Fig. 2). No significant difference between sucrose only and NS_dsRNA was observed.

### 3.2 Enhancement of stylin gene knockdown with gut nuclease-targeting dsRNA

To enhance the effectiveness of ST1 / ST2 knockdown by mitigating the premature degradation of ST1_ / ST2_dsRNA in the aphid gut, the coadministration of endonuclease-targeting Nuc1_dsRNA and/or Nuc2_dsRNA to suppress the expression, via RNAi of two nuclease genes, *Nuc1* and.

*Nuc2* (Ghodke et al., 2019), was conducted. The supplementation of ST1_dsRNA with Nuc1_dsRNA or Nuc2_dsRNA or combination of Nuc1+Nuc2_dsRNA was shown to significantly enhance gene knockdown of ST1 by 76% (*P <* 0.0001), 57% (*P <* 0.0001) and 43%, (*P <* 0.01), respectively (Fig. 3**A**). Likewise, the expression of the *ST2* was significantly downregulated with supplementation of ST2_dsRNA with Nuc1_dsRNA, Nuc2_dsRNA or Nuc1+ Nuc2_dsRNA by up to 84% (*P <*0.0001), 79% (*P* <0.0001) and 80% (*P* <0.0001), respectively when compared to sucrose control (Fig. 3**B**). Knockdown of ST1 and ST2 in this assay was contrasting in efficiency as the initial assays (**Refer** Fig. 2) with a non-significant reduction of 22% (vs 38.7%) observed for ST1 and a non-significant increase in knockdown observed of 41% (vs 25.7%) for ST2 highlighting inter-assay variation. Irrespective, defining the addition of nuclease-targeting dsRNA, a markedly enhanced knockdown of aphid Stylin genes, was observed with the supplementation of AD with Nuc1_dsRNA showing to be the most effective in enhancing reduction of *ST1* (76%) and *ST2* genes (84%), respectively.

### 3.3 Reducing ZYMV retention in the aphid stylet

To assess whether the knockdown of either *ST1* or *ST2* was able to reduce ZYMV particle retention in the aphid stylet, AD was prepared as previous except for the exclusion of Nuc2_dsRNA. After feeding on AD, the aphids were collected and transferred to ZYMV-infected leaves for 30 minutes AAP to acquire virus. ZYMV particle retention as measured by HC-Pro copy number in the aphids was observed to be reduced overall (Fig. 4). Aphids fed on AD containing ST1_dsRNA only showed a non-significant ∼38% (*P* = 0.372) reduction in ZYMV particle retention. However, upon the addition of Nuc1_dsRNA in the AD, a significant reduction of retained ZYMV particles by ∼84% (*P* < 0.0001) was observed (Fig. 4**A**). Similarly, aphids fed ST2_dsRNA showed a stronger and significant efficacy in reducing ZYMV retention by 68% (*P* = 0.0005). Likewise, upon the addition of Nuc1_dsRNA in the AD with ST2_dsRNA a significantly reduced ZYMV particle retention by up to 90% (*P* < 0.0001) was observed. Here, the knockdown of either *ST1* was shown to non-significantly reduce ZYMV particle retention however, upon the addition of Nuc1_dsRNA, particle retention was significantly reduced, presumably due to enhanced knockdown of receptor site ST1 and/ or ST2 proteins as observed in Fig. 3.

### 3.4 Mitigation of aphid transmitted ZYMV infection

Here we assessed whether reduced ZYMV particle retention translates to a reduction of ZYMV infection incidence *in planta*. Aphids were fed respective treatments and transferred to ZYMV-infected leaves for an AAP of < 30 mins to acquire virus. Five aphids were transferred onto assay plants and dispatched at 24 hours post placement. ZYMV transmission efficiency was determined by the infection incidence in assay plants at 10-days post-aphid- placement. Plants challenged by viruliferous aphids treated with sucrose only or NS_dsRNA diets showed an 83% infection rate, which is consistent with natural *M. persicae* mediated ZYMV infection incidence (Fig. 5**A**). Contrastingly, infection incidence was significantly reduced in the ST1+Nuc1_dsRNA group to 33%, in the ST2_dsRNA group to 39% and to only 22% in the ST2+Nuc1_dsRNA group (Fig. 5**A**).

Here, feeding of ST1_dsRNA only showed a limited effect on reducing the efficiency of aphid-mediated ZYMV transmission, which aligns with previous results of ZYMV retention (**refer** Fig. 4) leading evidence to ST1 not being the primary retention site for ZYMV. In contrast, ST2_dsRNA alone significantly reduced ZYMV particle retention (68%, P = 0.0005; Fig. 4) and produced the largest single-treatment reduction in transmission efficiency (53%; Fig. 5**B**), although the latter did not reach significance after correction. Together these results indicate that ST2 may be the more important target for disrupting ZYMV retention and, in turn, transmission. Supplementing AD treatments with Nuc1_dsRNA significantly reduced ZYMV transmission efficiency, in both ST1 (*P =* 0.0297) and ST2 (*P* = 0.0037) which is consistent with the results of the ZYMV retention assay (**refer** Fig. 4) and shows that the co-administration of Nuc1_dsRNA with ST1 or ST2_dsRNA significantly reduces ZYMV retention, compared to the administration of ST1 or ST2_dsRNA only.

## 4 Discussion

Growing concerns regarding insecticide resistance and the environmental and ecological impacts of chemical pesticides are driving the development of more specific and sustainable pest management strategies. Towards this goal, RNAi has emerged as a promising alternative, enabling sequence-specific silencing of target genes in insect pests without the respective consequences that manifest even with judicious pesticide use (Whyard et al., 2009; Christiaens et al., 2020). Of primary importance to effective RNAi applications against pest and pathogens is the identification of effective target genes. While many RNAi-based studies targeting insects pest such as aphids have focused on genes associated with survival, reproduction, and feeding behaviour the majority of primarily targeted genes in insects have been involved in maintaining essential physiological processes, such as proton flow (vATPase), insect development (cathepsin L), and regulatory functions (zinc finger protein (ZFP)) (Yan et al., 2020; Niu et al., 2019; Tariq et al., 2019; Xie et al., 2022). Silencing of these genes often results in high mortality or reduced fecundity, making them attractive targets for overall pest population control over time.

Conversely, RNAi applications targeting viral pathogens tend to focus on limiting *in planta* infection and replication. However, one critical aspect that such approaches have not directly addressed is the process of virus transmission, particularly for non-persistent viruses, where transmission occurs rapidly during brief probing events and is largely independent of long-term aphid survival. Here we proposed that disrupting genes directly involved in the virus-vector interaction represents a powerful, underutilised and novel approach for RNAi-based management of virus infection in plants.

The present study targeted stylin proteins (ST1 and ST2), which have emerged as the most likely candidates functioning as non-persistent virus-binding components in the aphid stylet (Liang & Gao, 2017; Webster et al., 2018). Evidence provided by Liang and Gao (2017) confirmed the positive interaction between *M. persicae* cuticle protein-4 (MPCP4) (corresponding to ST1 in later research articles) and the coat protein of CMV (direct attachment strategy) and showed a decrease in CMV acquisition by RNAi mediated knockdown of MPCP4 (Liang & Gao, 2017) and by Webster et al. (2018), who demonstrated that silencing of ST1 over ST2 reduced CaMV transmission (Webster et al., 2018).

In the application of RNAi to *M. persicae*, we achieved comparable knockdown efficiency of the ST1 and ST2 genes and demonstrated a moderate reduction in ZYMV-C19 particle reduction and transmission efficiency consistent with previous findings (Webster et al., 2018). However, the aim of this study was to investigate the potential exploitation of SIGS approach to disrupt the virus-vector interaction to limit virus retention, transmission and ultimately infection of crops as a field application. In this context our results were suboptimal and required a significant increase in knockdown efficiency, that could translate to significant reductions in virus particle retention, transmission efficiency and plant infection rates. Using this approach, we set out to achieve a 50% reduction in infection rate as a benchmark for practical disease management.

A major limitation of RNAi applications in most insects is the rapid degradation of dsRNA by nucleases present in the gut lumen. Several studies having identified dsRNases as the key barrier limiting RNAi efficiency in hemipteran insects (Cao et al., 2018; Christiaens et al., 2020; Ghodke et al., 2019; Jain et al., 2021; Jaubert-Possamai et al., 2007; Christiaens et al., 2014; Tayler et al., 2019). In this study, by co-administration of nuclease-targeting dsRNA with stylin-targeting dsRNA, we achieved a substantial enhancement of gene knockdown, increasing ST1 silencing efficiency from best case ∼39% to 76%. Likewise, with ST2, an increase from best case 41% silencing efficiency to 84% reduction was achieved. This increase is comparable to the increased knockdown efficiency of the endogenous melanin biosynthesis gene *yellow* of the Queensland fruit fly *B. tryoni* (Tephritidae) when AD was supplemented with dsRNAse targeting effectors (Tayler et al., 2019). This effect is most likely attributable to increased stability and persistence of dsRNA molecules in the aphid gut, a direct effect of reducing nuclease activity via the RNAi of RNAi approach employed. Using this approach, we theorise the concentration of intact Stylin dsRNA available for cellular uptake and processing is increased, thereby amplifying the RNAi response (Christiaens et al., 2014; Ghodke et al., 2019). It is also conceivable that suppression of dsRNases may facilitate increased systemic RNAi resulting from the increased dsRNA transport across gut tissues, a process that has been shown to be critical for effective gene silencing in insects (Christiaens et al., 2020). Therefore, the observed synergy between Stylin and nuclease dsRNA likely reflects a combination of improved dsRNA stability and availability, leading to increased uptake and enhanced intracellular processing.

The results of this study demonstrate that knockdown of stylin genes significantly reduces both ZYMV retention and transmission efficiency, supporting the evidence that stylins do play a primary role in non-persistent plant virus binding and retention within the aphid stylet. We showed by reducing ST1 and ST2 transcript abundance and presumably protein abundance/availability, a significant reduction in the retention of virus particles (84 - 90%) translating to a significant reduction in infection rate (60 – 73%) in test plants was achieved and exceeded our arbitrary set 50% threshold for practical disease management.

Interestingly, ST2 appeared to have a greater impact on ZYMV transmission than ST1, in contrast to previous findings in CaMV, where ST1 was identified as the primary functional receptor for HC-pro (P2) of CaMV (Webster et al., 2018) and potentially for CMV (Liang & Gao, 2017). The former study identified two 15 amino acid peptides of the C-terminus of ST1 (RYLASLPSTPEPKYQ) and ST2 (KLLASLPSTPEPQYQ) shared 80% identity, which are present on the surface of the pea aphid (*Acyrthosiphon pisum*) stylet and can be easily accessed (Webster et al., 2018). Also, competition binding of the *A. pisum* acrostyle between CaMV-P2 and the antibody targeting the peptide motif was observed, and the transmission efficiency of CaMV by *M. persicae* significantly reduced through RNAi silencing of *ST1*, while the knockdown of *ST2* did not cause a significant reduction in CaMV transmission. This then led to speculation that ST1 is the more abundant protein in the acrostyle. However, this conclusion was confounded by the minimal reduction in ST2 transcript knockdown (Webster et al., 2018).

An insight that our research provides evidence for is that ZYMV retention and transmission efficiency by *M. persicae* is more sensitive to the changes of *ST2* gene, when the efficiency of ST1 and ST2 knockdown are comparable. This discrepancy may reflect differences in virus-vector interactions between virus species, including variation in viral helper proteins and their binding affinities to the stylin proteins. Assuming that the conserved C-terminus motif of ST1 and ST2 proteins are the most likely binding site of the HC-Pro, our results suggest that the ST2 protein is the more prominent binding site for the ZYMV HC-pro (and potentially *Potyviridae* members) within the stylet of *M. persicae*. This is evidenced by the significantly reduced retention (ST1=38% vs ST2=68%) and transmission (ST1=13% vs ST2=53%) of ZYMV when ST2 is knocked down over ST1, irrespective of the inclusion of nuclease dsRNA. It is also conceivable that both ST1 and ST2 proteins interact with the viral bridging protein simultaneously, but with differing levels of interaction affinity contributing to the retention of ZYMV virus particles. Further investigation is required to determine whether ST1 and ST2 function as direct binding receptors for ZYMV HC-Pro and other potyviral members.

While artificial diet feeding provides a controlled system for evaluating antiviral RNAi efficacy, its direct application to field or glasshouse conditions is limited. For RNAi-based strategies mitigating transmission to be translated into practical crop protection solutions, effective delivery that enables dsRNA uptake under natural conditions are required. Previous studies have demonstrated the feasibility of plant-mediated RNAi, whereby transgenic plants expressing dsRNA targeting insect genes can suppress *M. persicae* populations (Pitino et al., 2011; Dong et al., 2022). Importantly, transgenic approaches to limit virus retention and transmission have precedent in a research context, as demonstrated by the silencing of the Ephrin receptor (Eph), to significantly reduce turnip yellows virus (TuYV) transmission by *M. persicae* (Mulot et al., 2018), with an important caveat here being TuYV is a virus which persistently infects its host.

In contrast to transgenic approaches, transdermal delivery systems have been developed to facilitate dsRNA penetration through the insect cuticle, providing an alternative route for RNAi induction. For example, nanocarrier/dsRNA/detergent-based formulation have been shown to significantly enhance dsRNA uptake, high target gene knockdown and population suppression of soybean aphids (*Aphis glycines*) (Zheng et al., 2019). Recent advances in SIGS RNAi have demonstrated that exogenously applied dsRNA can be taken up by plants and subsequently ingested by chewing insects such as Colorado potato beetle (*Leptinotarsa decemlineata*) (Rodrigues et al., 2021). In the context of this study, future work will be targeted to the combination of stylin-targeting dsRNA with more applicable delivery methods to provide a feasible, safe, and biocompatible strategy for reducing virus transmission in our agricultural growing systems.

In summary, our investigation showed that the candidate virus receptors, ST1 and ST2, can be efficiently knocked down by orally delivered dsRNA. Knockdown was enhanced by the co-administration of nuclease targeting dsRNAs, which translated to a significant reduction in ZYMV particle retention in aphids and reduced ZYMV transmission efficiency to plants. With the proven success of reducing ZYMV transmission efficiency through oral feeding, this study also highlights the importance of overcoming dsRNA degradation barriers in aphids with nuclease targeting being critical, in our opinion, to the effective development of biopesticide applications for hemipterans, irrespective of the delivery method. Coupled with our prior research targeting aphids (Ghodke et al., 2025) and viruses (Mitter et al., 2017; Jain et al., 2022) an integrated combinatorial approach targeting the virus’ ability to replicate in the plant (the virus-plant interaction), the vectors metabolic homeostasis to affect population decline overtime (the vector-plant interaction), bolstered by applications to disrupt of the ability of vectors to retain and transmit viruses (virus-vector interaction), as presented here, the development of SIGS RNAi based applications, effectively disrupting multiple critical virus-plant and virus-vector lifecycle interaction points, are developing as a promising, non-transgenic, and environmentally sustainable, viricidal crop protection application, for effective management of viral diseases in crops.

## Declaration of generative AI and AI-assisted technologies in the manuscript preparation process

During the preparation of this work the author(s) used Claude.ai in order to confirm statistical analysis and generate Fig. 2 – 5 and Supplementary Data 4. After using this tool, the authors reviewed and edited the content as needed and take full responsibility for the content of the published article.

## Funding

JL is supported by The University of Queensland HDR scholarship. KER is supported by Horticulture Innovation Australia (HIA) (AS22003) and Grains Research Development Corporation (GRDC) (UOQ2306-011RTX).

## CRediT authorship contribution statement

**Jingfeng Liang:** Writing – review & editing, Writing – original draft, Methodology, Investigation, Formal analysis, Conceptualization. **Amol Bharat Ghodke:** Conceptualization. **Stephen J Fletcher:** Writing – review & editing, Software, Conceptualization. **Narelle Manzie:** Writing – review & editing, Project administration. **Karl E Robinson:** Writing – review & editing, Writing – original draft, Supervision, Project administration, Funding acquisition, Formal analysis, Data curation, Conceptualization.

## Declaration of competing interest

The authors declare the following financial interests/personal relationships which may be considered as potential competing interests:

Dr Karl E Robinson reports financial support was provided by Horticulture Innovation Australia Limited. Dr Karl E Robinson reports financial support was provided by 10.13039/501100000980Grains Research and Development Corporation. If there are other authors, they declare that they have no known competing financial interests or personal relationships that could have appeared to influence the work reported in this paper.

## Data Availability

Data will be made available on request.

## Appendix Supplementary materials

Image, application 1

Image, application 2

Image, application 3

Image, application 4
