# Supplementary material for: RNAi targeting Myzus persicae (Hemiptera) stylet cuticle proteins reduces ZYMV transmission
Source: Virus Res. 2026 Aug 12;372:199784. doi: 10.1016/j.virusres.2026.199784 (PMC13524487; doi:10.1016/j.virusres.2026.199784)
Supplement: Supplementary file 1 [file mmc1.docx]

# **Supplementary data 1: dsRNA sequences of dsRNA effectors used in this study.**

# **Mp_ST1_dsRNA (250bp)** 181 – 430bp of *Myzus persicae* RR1 cuticle protein 1 mRNA, complete cds GenBank: DQ108938.1

Mp_ST1_T7_F	TAATACGACTCACTATAGGGagaagaagtcaggtacttgaagg

Mp_ST1_T7_R	TAATACGACTCACTATAGGGgtgctcaccgtcttactggt

TAATACGACTCACTATAGGGAGAAGAAGTCAGGTACTTGAAGGCTGGCCCAGAAGGACCCGTTTCAGTTGTCCAAGGAGCAGTGTCTTACGTGGCCCCAGACGGCCAGACCATCCAAACCGGCTACGTCGCCGACGAGAACGGTTACCAGCCGTACGGCGCTCACTTGCCCACTCCACCAGCAATCCCATTCGAGATCCAAGAGTCACTCAGATACCTCGCTTCTCTGCCCAGCACTCCTGAACCAAAATACCAGTAAGACGGTGAGCACGGGATATCACTCAGCATAAT

**Mp_ST2_dsRNA (278bp)** 191 – 468bp of *Myzus persicae* endocuticle structural glycoprotein SgAbd-4-like (LOC111031121) mRNAXM_022310930.1

Mp_ST2_T7_F	TAATACGACTCACTATAGGGgcccaaggtcccgaatctaag

Mp_ST2_T7_R	TAATACGACTCACTATAGGGtcagctgggattggtggtga

TAATACGACTCACTATAGGGGCCCAAGGTCCCGAATCTAAGGCCACCATCCTGCGTCAAGAACAAGAAGTCAACTTCGACGGAAACTTCAAGAACAAATTCGAAACCGATAACGGCATCAAACAAGAAGAAGTCGGTTACGCAAAAGCTGGACCCGAAGGCCCCATCTCAATTGTCCAGGGAGCAAATTCCTACCAAGCCCCCGATGGTTCAATCATCTCACTCGGTTACACCGCTGACGAGTTCGGTTACCATCCATACGGCGCCCATTTGCCGACTTCACCACCAATCCCAGCTGAGGGATATCACTCAGCATAAT

**Mp_Nuc1_dsRNA** (328 bp). 559 – 887bp of *Myzus persicae* uncharacterized LOC111042657 transcript variant X1, mRNA XM_022327342

Mp_NUC1_T7_F	TAATACGACTCACTATAGGGtcctccgacgcgttggtcac

Mp_NUC1_T7_R	TAATACGACTCACTATAGGGtgttgccgtacagctctttg

TAATACGACTCACTATAGGGTCCTCCGACGCGTTGGTCACGTGCGTCGGCGGCGACGTGCTCGCGTACCGAGGCCAGACGTACCGGTACGCCGCCTTCCAGTGCGACGGCATGCCCAAGTCGGAGCTCCGCGTCACCGACGAAGTGTGCCAGCCGGCCAACTACACCGTGGCCGTGGTCGGTTTCCGGACGGATCGCGCGTTCCTCCGGCTGTACGGCATGTGCTTCGACAAGTCGACCAAGAACAGCCTGTACACTTGGTACGACGCCCGGTCGCCGTACTACGACAACCACCAGAAGTATAGCAAGCGACCGTCGTTCAACAAAACCAAAGAGCTGTACGGCAACACGGGATATCACTCAGCATAAT

# **Mp_Nuc2_dsRNA** (304bp) (136 – 440bp of *Myzus persicae* nonspecific endonuclease 2 mRNA, complete cds GenBank: MN257577.1

Mp_NUC2_T7_F	TAATACGACTCACTATAGGGgatggccttgtggccaataat

Mp_NUC2_T7_R	TAATACGACTCACTATAGGGgctaaatgcccacggtcaaa

TAATACGACTCACTATAGGGGATGGCCTTGTGGCCAATAATCAGGAACTAATTGTTAATAACGAACAGCGAATATCTCAGATCATGAAATATGGATTTCCGAGTTTGGATAATGTACGTTCGTATAAGAATTTTGTTCTTTCTTATGATCAAAGGAATCGTATACCCCATTGGGTTCTAGAACATCTCACACCAGAAAGCGTACAATATAATCCTAATGTAGATAGATCGTCATGTGAATTTTATGAAGATAACTCATTTCATGAGTTTTTTCGAGCTACAAATAAAGATTATAAAAATAGTGGTTTTGACCGTGGGCATTTAGCGGGATATCACTCAGCATAAT

**NS_dsRNA** (300bp) – non-specific randomly generated sequence. Purchased from AgroRNA.

CACAUUUCUCGAAUGUCCAUCUGAGGGGGCUACUCUUUGCUUUAAUUUGUUCGGAACUACAACGUAAAGGUCGGUUAAAACAAGGUCACCCUUGGUUAGACUACGUGAGGUCGAAUGUUGAAACGAUCCACGCUCAAUGCAUGUUUGACUACAUGUAUUGCACAUACCUUAAAUUUAGAAAUAAACGACAAGCACGCGAAGUCUGUUUUCUCUUCACUAAAUCAGCUUCCAAUGUAUAUUUCUGGUAUAAAUAUGGAUUAGUAAUUCGACCAGGUAAUGAGACUCAGGGGAACUUACCGA
