# Supplementary material for: RNAi targeting Myzus persicae (Hemiptera) stylet cuticle proteins reduces ZYMV transmission
Source: Virus Res. 2026 Aug 12;372:199784. doi: 10.1016/j.virusres.2026.199784 (PMC13524487; doi:10.1016/j.virusres.2026.199784)
Supplement: Supplementary file 2 [file mmc2.docx]

# Supplementary data 2: Primer Efficiency

ST1

| Log10 value | Ct Value |
| --- | --- |
| 0 | 25.502 |
| -1 | 28.9324 |
| -2 | 32.6284 |
| -3 | 35.8947 |

ST2

| Log10 Value | Ct Value |
| --- | --- |
| 0 | 19.391 |
| -1 | 22.6178 |
| -2 | 26.0784 |
| -3 | 29.2428 |

RPL7

| Log10 Value | Ct Value |
| --- | --- |
| 0 | 19.7072 |
| -1 | 22.7128 |
| -2 | 26.346 |
| -3 | 28.8485 |

**Supplementary Data 2**: **Real-time qPCR primer efficiency for stylin-001 (ST1), stylin-002 (ST2), and ribosomal protein L7 (RPL7) genes.** Ten-fold serial dilutions of *M. persicase* aphid total RNA from 100 to 0.1 (ng) were amplified in 4 points × 2 technical replicates. Standard curve of ST1 primers (Ct vs log10 input) yielded slope = –3.4874, y-intercept = 25.508, R² = 0.9995, corresponding to efficiency = 93.5%. Standard curve of ST2 primers (Ct vs log10 input) yielded slope = –3.3016, y-intercept = 19.380, R² = 0.9997, corresponding to efficiency = 100.9%. Standard curve of RPL7 primers (Ct vs log10 input) yielded slope = –3.1057, y-intercept = 19.745, R² = 0.9955, corresponding to efficiency = 109.9%.
