# Supplementary material for: RNAi targeting Myzus persicae (Hemiptera) stylet cuticle proteins reduces ZYMV transmission
Source: Virus Res. 2026 Aug 12;372:199784. doi: 10.1016/j.virusres.2026.199784 (PMC13524487; doi:10.1016/j.virusres.2026.199784)
Supplement: Supplementary file 3 [file mmc3.docx]

# Supplementary data 3: ZYMV HC-Pro Standard curve for virus titre determination

HC-Pro Standard Curve

Ct

| Log10 (HC-Pro Copy number) | Ct |
| --- | --- |
| 10.2183 | 16.5413 |
| 9.21826 | 19.494 |
| 8.21826 | 22.6799 |
| 7.21826 | 26.3441 |
| 6.21826 | 29.3901 |
| 5.21826 | 32.9418 |

$$\mathbf{EQ1}: Copy number= \frac{Quantity of RNA \left( \mathrm{ng} \right) x Avogadro constant:6.022 x {10}^{23}}{RNA length \left( 552 bp \right) x 1 x {10}^{9} x average weight of dsRNA per bp (660 g/mol)}$$

**Supp Data 3: Real-time qPCR amplification of ZYMV-HC-Pro gene for absolute quantification of ZYMV viral retention in *M. persicase* aphids.** Ten-fold serial dilutions of ZYMV-HC-Pro dsRNA from 10 to 0.0001 (ng) were amplified in 6 points × 4 technical replicates. Standard curve of HC-Pro dsRNA amplification (Ct vs log10 copy number per mL) yielded slope = –3.2959, y-intercept = 56.552, R² = 0.9991. Copy number per mL calculated using the formula: Copy number per mL = [Quantity of RNA (ng) x Avogadro constant:6.022 x 10^23] / [RNA fragment length (552 bp) x 1 x 10^9 x average molecular weight of dsRNA per bp (660 g/mol)].
