# Supplementary material for: RNAi targeting Myzus persicae (Hemiptera) stylet cuticle proteins reduces ZYMV transmission
Source: Virus Res. 2026 Aug 12;372:199784. doi: 10.1016/j.virusres.2026.199784 (PMC13524487; doi:10.1016/j.virusres.2026.199784)
Supplement: Supplementary file 4 [file mmc4.docx]

**Supplementary data 4: NS_dsRNA Off-Target Control — Statistical Analysis**

**Data Structure**

Two stylet gene targets (ST1 and ST2), each assessed across two biological trials, with three technical replicates per treatment. Treatments were: Sucrose (vehicle control), NS 300, NS 450, and NS 600 ng/μL. Data are expressed as 2⁻ᐩᐩCt relative expression values, where 1.0 = no change.

**Descriptive Statistics (Trials Pooled, n = 6 per group)**

| **Gene** | **Group** | **Mean** | **SEM** | **95% CI** |
| --- | --- | --- | --- | --- |
| ST1 | Sucrose | 1.037 | 0.122 | 0.72 – 1.35 |
| ST1 | NS 300 | 2.124 | 0.460 | 0.94 – 3.31 |
| ST1 | NS 450 | 1.880 | 0.268 | 1.19 – 2.57 |
| ST1 | NS 600 | 2.374 | 0.443 | 1.24 – 3.51 |
| ST2 | Sucrose | 1.039 | 0.136 | 0.69 – 1.39 |
| ST2 | NS 300 | 1.989 | 0.431 | 0.88 – 3.10 |
| ST2 | NS 450 | 2.136 | 0.362 | 1.21 – 3.07 |
| ST2 | NS 600 | 1.915 | 0.458 | 0.74 – 3.09 |

**Statistical Tests**

One-way ANOVA was used to assess overall group differences, followed by Bonferroni-corrected pairwise t-tests (6 comparisons) to control family-wise error rate.

| **Gene** | **F-statistic** | **p-value** |
| --- | --- | --- |
| ST1 | 2.73 | 0.071 |
| ST2 | 1.81 | 0.179 |

All pairwise comparisons between NS concentrations and Sucrose were non-significant after Bonferroni correction (all p_adj > 0.05, annotated as "ns"). Critically, no significant differences were detected between any of the three NS_dsRNA concentrations (NS 300 vs 450 vs 600), with all inter-NS p_adj values approaching 1.0.

**Interpretation**

These results support the validation of NS_dsRNA as a robust off-target control across all three concentrations tested:

1. No significant gene expression changes were detected for either ST1 or ST2 in response to NS_dsRNA at any concentration, compared to Sucrose control.
2. No dose-dependent effect was observed — expression levels across NS 300, 450, and 600 ng/μL were statistically indistinguishable from one another.
3. The elevated means in the NS groups (approximately 2× relative to Sucrose) are driven by high variance between replicates/trials rather than a true biological signal — the wide confidence intervals and non-significance on ANOVA confirm this.
4. The Sucrose control is tightly distributed around 1.0 as expected, confirming the ΔΔCt normalisation is working correctly.

**Conclusion:** NS_dsRNA does not alter expression of either stylet gene target across the tested concentration range (300–600 ng/μL), validating its use as a negative/off-target control for the subsequent stylet gene knockdown experiments.
